# Pelvic Floor Muscle Exercises as a Treatment for Urinary Incontinence in Postmenopausal Women: A Systematic Review of Randomized Controlled Trials

**DOI:** 10.3390/healthcare11020216

**Published:** 2023-01-11

**Authors:** María Paz López-Pérez, Diego Fernando Afanador-Restrepo, Yulieth Rivas-Campo, Fidel Hita-Contreras, María del Carmen Carcelén-Fraile, Yolanda Castellote-Caballero, Carlos Rodríguez-López, Agustín Aibar-Almazán

**Affiliations:** 1 Department of Health Sciences, Faculty of Health Sciences, University of Jaén, 23071 Jaen, Spain; 2 Faculty of Health Sciences and Sport, University Foundation of the Área Andina-Pereira, Pereira 660004, Colombia; 3 Faculty of Human and Social Sciences, University of San Buenaventura-Cali, Santiago de Cali 760016, Colombia; 4 Gimbernat-Cantabria School of Physiotherapy, University of Cantabria, 39005 Santander, Spain

**Keywords:** urinary incontinence, pelvic-floor-muscle exercises, postmenopausal women, systematic review

## Abstract

Women frequently suffer from urinary incontinence due to atrophic changes in the urogenital tract. Recommended conservative treatment includes evaluation of pelvic-floor strength and the functional use of pelvic-floor-muscle (PFM) training. Following the PRISMA 2020 guidelines, a search was conducted in the electronic databases PubMed, Web of Science, and Scopus for articles with at least one group performing PFM exercises in post-menopausal women with urinary incontinence. Eight articles were included, and each study had at least one group of PFM exercise-based intervention alone or combined. The volume or duration, frequency, and number of sessions were heterogeneous. All the studies reported significant differences in favor of PFM exercise in strength, quality of life, and/or severity of urinary incontinence. PFM exercise is a highly recommended intervention to treat urinary incontinence in postmenopausal women. However, more research is needed to establish specific factors such as dose–response relationships and to standardize methods for measuring effects.

## 1. Introduction

Urinary incontinence was defined in 2003 as the complaint of any involuntary leakage of urine [1]. This condition is associated with risk factors such as pelvic-floor-muscle (PFM) deficits, pelvic surgery, prolapse, urinary-tract infections, obesity, smoking, constipation, diabetes mellitus, high-impact physical exercise, being female, increasing age, parity, and menopause [2,3].

Postmenopausal women frequently suffer from urinary incontinence as a result of increased intra-abdominal pressure, such as sneezing, coughing, jumping, laughing, or sexual relations [4]. Estrogen deficiency at this stage of the life cycle generates atrophic changes in the urogenital tract and vaginal and periurethral tissues [5], and has been associated with involuntary urine loss due to stress and increased urinary urgency and frequency [6]. Despite this association, there has been no evidence of improvement with hormonal management [7]. 

Among the options based on non-invasive and non-pharmacological intervention are therapeutic targeted exercise such as PFM training, which focuses on improving the function, muscle tone, strength, coordination, and endurance of the pelvic-floor musculature [8]. Other active treatment techniques are Kegel exercises, which focus on enhancing the strength and improving the function of the PFM [9], or pelvic-floor contraction exercises coupled with coactivation of the trunk-stabilizing muscles [10].

Conservative treatment, recommended as first line by the International Continence Society, includes assessment of pelvic-floor strength and functional use of PFM training [11]. The success of this intervention lies in the achievement of increased contraction and holding strength, coordination, speed, and endurance of the pelvic-floor musculature to keep the bladder elevated during demanding activities. Likewise, PFM training allows adequate urethral closure pressure to be maintained and supports and stabilizes the pelvic organs [12]. For postmenopausal women who receive regular supervision, it has been observed that they are more likely to comply and report a decrease in urinary incontinence than women who perform PFM training with little or no supervision [13]. 

Other systematic reviews related to exercise in this population can be found in the literature; however, they focused on determining the effects of exercise on quality of life or on comparing different interventions with this type of training on multiple variables associated with the pathology [14,15]. Therefore, the aim of the present review was to perform a systematic review of randomized controlled clinical trials that evaluated the effect of targeted PFM exercise in postmenopausal women for the treatment of urinary incontinence.

## 2. Materials and Methods

This systematic review was performed following the guidelines of the PRISMA statement (Preferred Reporting Items for Systematic reviews and Meta-Analysis) [16]. The pre-specified protocol was registered in PROSPERO under the code CRD42022373488.

### 2.1. Eligibility Criteria

Articles were selected according to the following criteria: clinical trial, randomized control trial with objective measures of urinary incontinence before and after an exercise-based intervention in postmenopausal women. Regarding the intervention, articles in which the PFM training method was used for the treatment of urinary incontinence during the postmenopausal period were included.

### 2.2. Information Sources

Data collection took place from October to November 2022 by consulting the following databases: Pubmed (MEDLINE), Scopus, and Web of Science. 

### 2.3. Search Strategy

The keywords used were (“postmenopausal period” OR “postmenopausal” OR “postmenopausal women”) AND (“diurnal enuresis” OR “enuresis” OR “daytime wetting” OR “daytime urinary incontinence” OR “urinary incontinence”) AND (“pelvic floor muscle training” OR “pelvic floor exercises” OR “pelvic floor muscle exercise” OR “pelvic floor muscles”) AND (“severity” OR “frequency of urination” OR “urinary frequency” OR “urination behaviors” OR “frequency of micturition” OR “micturition” OR “quality of life” OR “mental health” OR “depression” OR “sexual activity”).

### 2.4. Selection Process

The search results were exported to the Rayyan QCRI application (https://rayyan.qcri.org/welcome accessed on 15 November 2021) [17]. Two blinded independent researchers conducted the literature review and decided on the inclusion of the articles separately. The pre-selection of the studies was performed based on reading of the title and abstract. Subsequently, the pre-selected articles were read in full text and the articles that met the criteria were included. In case of discrepancies, a third author resolved them.

### 2.5. Data-Collection Process

The main variable of this review was the objective measurement of urinary incontinence, mainly in terms of strength, quality of life, and severity of the incontinence. We included information on the authors, the year of publication, the country of publication, and the country in which the study was conducted; likewise, we collected the type, duration or volume, frequency, number of sessions, and number of weeks of the interventions performed, as well as the follow-up time and the results obtained in each measurement.

### 2.6. Assessment of Methodological Quality

The methodological quality of the articles included in this review was assessed using the PEDro scale [18], with a maximum score of 10 points, as the first item (“eligibility criteria”) is not used in the final score calculation. Each item can be answered as either “Yes” (1 point) or “No” (0 points) [19]. A score between 0 and 3 was considered “Poor” quality, 4–5 “Fair,” 6–8 “Good,” and >9 “Excellent” [20]. The scores were consulted in the PEDro database; when scores were not found, two authors evaluated the methodological quality of articles, and in situations where a discrepancy was generated, it was resolved by a third author. 

## 3. Results

### 3.1. Selection of the Studies

**Figure 1:**
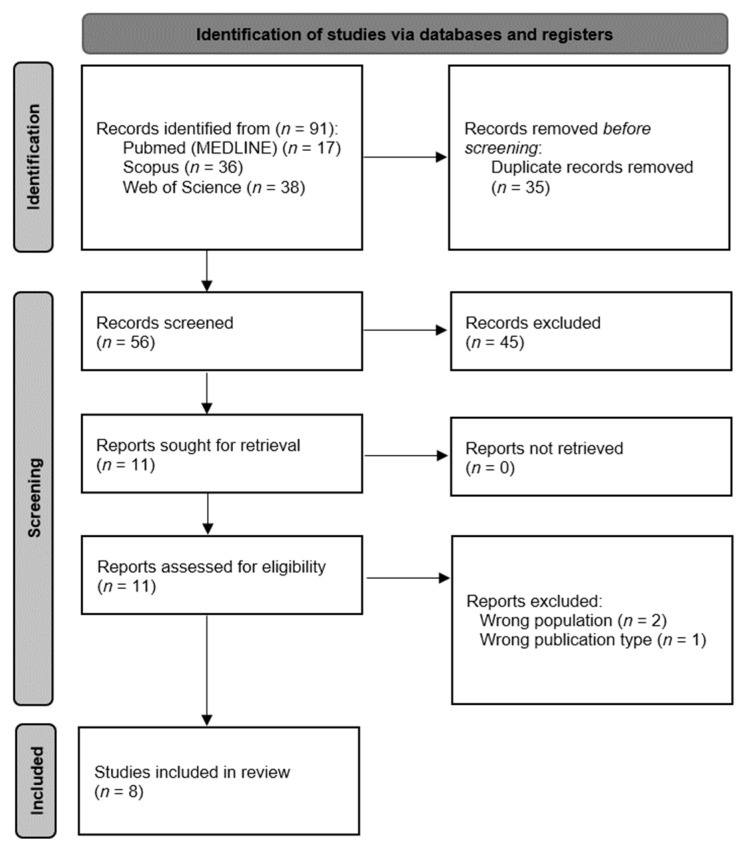
Flow diagram of the study-selection process.

The database search resulted in a total of 91 articles, which were revised to identify duplicates, discarding 35 and leaving 56 unique articles. After a title-and-abstract screen, 11 potentially eligible articles remained. Finally, only eight articles [21,22,23,24,25,26,27,28] met the eligibility criteria established for this review (Figure 1).

### 3.2. Methodological Quality

Methodological quality was assessed using the PEDro scale. The scores of seven of the articles [21,22,23,24,25,26,27] were obtained from the PEDro website, whereas the remaining article [28] was calculated manually. Seven of the articles [22,23,24,25,26,27,28] included in this review presented ”Good” methodological quality, and only one [21] had “Fair” methodological quality (Table 1).

### 3.3. Characteristics of the Studies

The articles included in this systematic review were all randomized controlled clinical trials published in Switzerland [21,25], the United Kingdom [22,23,24], the United States [26,27], and Poland [28]; however, the studies were conducted in countries other than those in which they were published, such as Turkey [21], Australia [22], Brazil [23,24,25,27], Canada [26], and Egypt [28].

A total of 376 postmenopausal women aged 60.31 ± 6.73 years participated in the included studies. Out of the overall population, 196 postmenopausal women were part of the groups that received PFM exercise-based treatments (Table 2). 

### 3.4. Study Intervention

Every study [21,22,23,24,25,26,27,28] included at least one group with a PFM exercise-based intervention. Six studies [21,22,23,24,25,27] performed PFM exercise-based interventions only, whereas Sran et al. [26] combined it with physiotherapy and Ghoniem et al. [28] with Pilates. 

Although all the interventions included PFM exercises, the prescription of the volume or duration, frequency, and number of sessions was heterogeneous. Regarding frequency, one study proposed an intervention program with only one intervention per week [26], five studies proposed two sessions per week [23,24,25,27,28], one study maintained three sessions per day but did not specify the number of days per week [21], and one study did not specify either sessions per day or per week. The number of sessions ranged from 8 [27] to 12 [23,24,26] to 24 [25,28]; however, two studies did not specify the number of sessions [21,22].

Concerning the volume or duration of the exercises, four of the studies [23,24,26,27] dosed the exercises based on time, with sessions lasting from 20 min to 60 min. On the other hand, three studies [21,25,28] dosed the exercise based on the number of contractions and positions used, ranging from 10 contractions and one single position [21], to four positions and 10 contractions in each [25], and to positions with up to 52 contractions after the adaptation period [28].

### 3.5. Study Results

All the articles found a significant difference in favor of the PFM-exercise intervention in at least one variable related to the strength of this musculature, severity of incontinence, and/or quality of life [21,22,23,24,25,26,27,28]. In addition, when PFM exercises were applied in combination with other interventions, no significant differences were observed with the groups that did PFM exercises alone [28]. 

Similarly, to the interventions, the outcomes measured remained heterogeneous. Statistically significant changes (*p* < 0.05) were observed in the 1 h pad test [21,26], perineometry [21], PFM strength with digital palpation [21], incontinence frequency [21], stress test [22], urinary leakage [23,24], PFM pressure [23,24], incontinence impact [23,24], gravity measures [23,24], urinary-incontinence severity [25], number of leakage episodes [26], Urogenital Distress Inventory total score [26], precontraction [27], initial electromyographic baseline [27], duration of endurance contraction [27], maximum voluntary contraction [27], International Consultation Incontinence Questionnaire—Short Form [27], squeeze vaginal pressure [28], and the Urinary Incontinence Scale [28]. 

## 4. Discussion

The present systematic review aimed to determine the effects of PFM exercises in the treatment of urinary incontinence in postmenopausal women. The review included eight randomized controlled trials that met the selection criteria [21,22,23,24,25,26,27,28]. After analysis of the studies, scientific evidence was found to support the use of PFM training as an effective intervention for incontinence in the studied population.

Several risk factors predispose to the development of urinary incontinence in women, such as high parity, history of vaginal deliveries, and menopause [29,30,31]. In addition, obesity and aging are also important variables for the development of urinary incontinence independent of sex [32]. Within the eight articles included in this review, six studied overweight postmenopausal women (BMI > 25 Kg/m^2^–<30 Kg/m^2^) [22,23,24,25,27,28], one included postmenopausal women with normal weight [26], and finally, one article did not report BMI [21]. However, regardless of the BMI of the participants, the effects were statistically significant in all studies, which is in agreement with the systematic review made by Woodley et al. [33], who also conducted studies with varied BMI populations and observed favorable effects in all articles.

From the eight articles included in this review, 5 = five [21,22,23,27,28] focused on stress urinary incontinence only, two [24,25] included patients with stress or urgency urinary incontinence, and just one [26] of the articles included patients with stress, urgency, or mixed urinary incontinence; however, the effects of PFM training were statistically significant irrespective of this factor.

All studies used different measurement techniques to assess strength, quality of life, and the severity and prevalence of the urinary incontinence. Regarding strength, six articles [21,23,24,25,27] found statistically significant favorable changes in all of them. This is congruent with the findings of Alouini et al. [34], who reported similar results regarding the improvement of strength through PFM exercises in women. Strength production is mainly due to two factors: muscle-fiber trophism and motor-unit recruitment capacity. Current evidence suggests that changes in strength, at least during the first 8 weeks of a training protocol, are mainly caused by an increase in motor-unit recruitment capacity [35,36]. The protocols included in this review that found favorable results in terms of strength ranged from 6 to 12 weeks, finding in neural adaptations an explanation for their results. Additionally, one study evaluated the long-term effects [24], reporting that after 6 months, the strength gain decreased, however the change was not large enough to reach baseline.

The prevalence and severity of urinary incontinence was evaluated in seven of the studies [21,22,23,24,25,26,28], with statistically significant changes observed, both acutely and chronically, in favor of the groups that performed PFM exercise. The most prevalent ways to measure this variable were the pad test [21,26] and the amount or number of urinary leakages [23,24,26]. These results are similar to those obtained by other authors who determined the effects of PFM training in other population groups [33,37,38,39].

Quality of life was assessed in six [21,23,24,25,26,27] out of the eight studies included in this review, using different instruments such as the Social Activity Index [21], the International Consultation Incontinence Questionnaire—Short Form [25,27], the Incontinence Impact Questionnaire [26], and three domains (general health, incontinence impact, and gravity) of the King’s Health Questionnaire [23,24]. Only one of the studies [25] did not show statistically significant changes in this variable, mainly because the population of the intervention and control groups were not balanced from baseline (intergroup difference at baseline *p* = 0.03). Usually, patients with urinary incontinence present discomfort, low self-esteem, mood deterioration, and a feeling of helplessness, which generates an important psychological impact that ends up affecting the quality of life of the patient [37,40,41,42]. This is why interventions that generate a decrease in urinary incontinence are associated with an improvement in quality of life [43]. 

This systematic review is the first to evaluate the effects of PFM exercises in postmenopausal women with urinary incontinence; however, it has several limitations, and the results should be interpreted with discretion. The great heterogeneity in the exercise prescription does not allow an optimal prescription of the intervention to be established. In addition, no study was carried out in a European population; hence, a geographic bias was observed. Finally, it was not possible to calculate the size of the effects through a meta-analysis due to the great variety in the variables and the instruments and measurement techniques used by the different authors. 

## 5. Conclusions

PFM exercise is a highly recommended intervention for treating urinary incontinence in postmenopausal women, whether it is applied alone or in combination with other interventions. Although the studies included in this review suggest that PFM training is effective regardless of the type of urinary incontinence, the current evidence is insufficient to be certain. Additionally, it is necessary to establish specific criteria for prescribing PFM exercises and measuring their results. More research in this field is needed, focused mainly on establishing the dose–response relationship of this intervention and on standardizing the methods of measuring the effects. 

## Figures and Tables

**Table 1:** Methodological quality of the articles included.

Article	1	2	3	4	5	6	7	8	9	10	11	Total
Aksac et al., 2003 [21]	Y	Y	Y	Y	N	N	N	N	N	Y	Y	5
Sherburn et al., 2011 [22]	Y	Y	Y	Y	N	N	Y	Y	Y	Y	Y	8
Pereira et al., 2012 [23]	Y	Y	Y	N	N	N	N	Y	Y	Y	Y	6
Pereira et al., 2013 [24]	Y	Y	Y	Y	N	N	N	Y	N	Y	Y	6
Flávia et al., 2015 [25]	Y	Y	Y	Y	N	N	Y	Y	Y	Y	Y	8
Sran et al., 2016 [26]	Y	Y	Y	Y	N	N	Y	Y	Y	Y	Y	8
Bertotto et al., 2016 [27]	Y	Y	Y	Y	N	N	N	Y	N	Y	Y	6
Ghoniem et al., 2022 [28]	Y	Y	Y	Y	N	N	N	Y	N	Y	Y	6

Items: 1 = eligibility criteria; 2 = random allocation; 3 = concealed allocation; 4 = baseline comparability; 5 = blind subjects; 6 = blind therapists; 7 = blind assessors; 8 = adequate follow-up; 9 = intention-to-treat analysis; 10 = between-group comparisons; 11 = point estimates and variability; Y = Yes; N = No.

**Table 2:** Characteristics of the included studies.

Author and Year	Sample CG/IG	Control Group	Type of Urinary Incontinence	Intervention Group					
				Population Characteristics	Intervention Type	Variable Observed–Initial Measure	Modifications over Time		
Aksac et al., 2003 [21]	10/20	No treatment	Stress urinary incontinence	Age: 52.5 ± 7.9 Body weight: 59.4 ± 6.1 Parity: 2.8 ± 0.5	T: PFM exercises V: 10 contractions/session F: 3 sessions/day S: Not specified	1 h pad test, g 9.9 (SD = 2.5) Perineometry, cmH_2_O 20.3 (SD = 6.2) PFM strength with digital palpation 3.5 (SD = 0.5) Incontinence frequency 2.3 (SD = 0.7) SAI 4.5 (SD = 0.3)	8 weeks 1 h pad test, g 2.1 (SD = 0.4) * Perineometry, cmH_2_O 37.5 (SD = 8.7) * PFM strength with digital palpation 4.8 (SD = 0.4) * Incontinence frequency 3.5 (SD = 0.5) * SAI 7.5 (SD = 1.2) *	—	—
Sherburn et al., 2011 [22]	41/43	Bladder training	Stress urinary incontinence	Age: 71.6 ± 4.73 BMI: 27.6 ± 3.88 Parity: 3.2 ± 1.6	T: PFM exercises V: Not specified F: Not specified S: Not specified	Stress test—cough (g) 0.8 (IQR = 4.9) Stress test—brace/cough (g) 0.2 (IQR = 2.2)	1 month Stress test—cough 0.9 (IQR = 2.0) Stress test—brace/cough 0.2 (IQR = 0.5)	3 months Stress test—cough 0.6 (IQR = 2.8) Stress test—brace/cough 0 (IQR = 0.5) *	5 months Stress test—cough 0.1 (IRQ = 1.5) * Stress test—brace/cough 0 (IRQ = 0.3) *
Pereira et al., 2012 [23]	14/15	No treatment	Stress urinary incontinence	Age 63 ± 10.73 BMI: 25.65 ± 2.79 Parity: 2.26 ± 1.09	T: PFM exercises D: 40 min F: 2 times/week S: 12 sessions	Urinary leakage (g) 3.70 (SD = 4.35) PFM pressure 12.55 (SD = 9.20). General health 33.34 (SD = 18.09) Incontinence impact 55.82 (SD = 39.32) Gravity measures 41.33 (SD = 25.47)	6 weeks Urinary leakage (g) 0.19 (SD = 0.27) * PFM pressure 37.38 (SD = 18.18) * General health 23.33 (SD = 6.45) Incontinence impact 7.69 (SD = 14.6) * Gravity measures 5.91 (SD = 6.26) *	12 weeks Urinary leakage (g) 0.29 (SD = 0.31) * PFM pressure 35.22 (SD = 18.96) * General health 30.01 (SD = 16.90) Incontinence impact 17.76 (SD = 24.7) * Gravity measures 15.11 (SD = 23.0) *	—
Pereira et al., 2013 [24]	13/13	No treatment	Stress or urgency urinary incontinence	Age 62 (51–85) BMI: 25.7 (24.3–31.8) Parity: 2.0 (0–4)	T: PFM exercises D: 40 min F: 2 times/week S: 12 sessions	Urinary leakage (g) 1.9 (1.0–15.2) * PFM pressure (cmH_2_O) 10.7 (2.7–43.3) General health 25.0 (0–75) Incontinence impact 33.3 (0–100) Gravity measures 46.7 (0–73.3) *	3 months Urinary leakage (g) 0.1 (0–0.9) * PFM pressure (cmH_2_O) 37.3 (15.3–60) * General health 25.0 (0–50) Incontinence impact 0.0 (0–0) * Gravity measures 0.0 (0–7) *	6 months Urinary leakage (g) 0.1 (0–1.2) * PFM pressure (cmH2O) 15.3 (7.3–60) * General health 25.0 (0–25) Incontinence impact 0.0 (0–33) * Gravity measures 0.0 (0–20) *	—
Flávia et al., 2015 [25]	41/47	No treatment	Stress or urgency urinary incontinence	Age 52.9 ± 4.1 BMI: 28.5 ± 5.4 Vaginal births: 1.1 ± 1.4	T: PFM exercises V: 4 positions with 10 voluntary maximal contractions each F: 2 times/week S: 24 sessions	PFM strength (cmH_2_O) 38.5 (SD = 23.6) Prevalence of UI, n/N (%) 21/47 (44.7%) UI severity (0 to 21) 3.8 (SD = 5.0) *	12 weeks PFM strength (cmH_2_O) 44.7 (SD = 24.0) * Prevalence of UI, n/N (%) 17/47 (36.2%) * UI severity (0 to 21) 1.9 (SD = 2.9)	—	—
Sran et al., 2016 [26]	24/24	Osteoporosis education	Stress, urgency, or mixed urinary incontinence	Age 66.17 ± 6.66 BMI: 24.69 ± 3.93 Parity: 1.35 ± 1.15	T: Physical therapy + PFM training D: 30–60 min F: 1 time/week S: 12 sessions	# of leakage episodes 8.00 (4.00–10.50) Pad test (weight, g) 6.50 (3.00–25.50) UDI total score 113.07 (75.85–137.41) IIQ total score 53.06 (23.33–88.13) Self-perceived efficacy 0.51 (0.38–0.68)	3 months # of leakage episodes 2.00 (0.00–6.00) * Pad test (weight, g) 3.50 (2.00–8.50) UDI total score 62.88 (34.37–103.98) * IIQ total score 9.72 (0.00–36.19) * Self-perceived efficacy 0.72 (0.50–0.83) *	12 months # of leakage episodes 2.00 (0.00–5.75) * Pad test (weight, g) 2.50 (1.00–3.75) * UDI total score 66.29 (30.50–90.91) * IIQ total score 6.95 (0.00–26.39) Self-perceived efficacy 0.64 (0.51–0.76)	—
Bertotto et al., 2016 [27]	15/15	No treatment	Stress urinary incontinence	Age 59.3 ± 4.9 BMI: 27.7 ± 3.6 Number of pregnancies: 2.3 ± 1.3	T: PFM exercises D: 20 min F: 2 times/week S: 8 sessions	Precontraction 0.13 (SD = 0.9) Initial EMG baseline (μv) 14.7 (SD = 4.4) Final EMG baseline (μv) 15.5 (SD = 3.3) DEC (s) 1.66 (SD = 2.55) MVC (μv) 10.3 (SD = 2.11) ICIQ-SF quality of life score 11.1 (SD = 2.9)	6 weeks Precontraction 0.67 (SD = 0.12) * Initial EMG baseline (μv) 16.3 (SD = 2.9) * Final EMG baseline (μv) 15.9 (SD = 2.4) DEC (s) 6.8 (SD = 2.01) * MVC (μv) 20 (SD = 5.21) * ICIQ-SF quality-of-life score 4.3 (SD = 3.2) *	—	—
Ghoniem et al., 2022 [28]	15/15	Same program without Pilates	Stress urinary incontinence	Age 55.13 ± 4.48 BMI: 26.86 ± 1.92 Parity: not reported	T: PFM exercises + Pilates D: 3 positions with up to 52 contractions F: 2 times/week S: 24 sessions	Squeeze vaginal pressure CG: 18.1 (SD = 6.25)/ IG: 18.33 (SD = 6.45) Urinary-incontinence scale CG: 11.46 (SD = 1.95) IG: 10.93 (SD = 2.08)	12 weeks Squeeze vaginal pressure CG: 23.33 (SD = 9.29) * IG: 26.66 (SD = 9.29) * Urinary-incontinence scale CG: 10.33(SD = 2.19) * IG: 9.06 (SD = 1.62) * No significant difference between groups	—	—

CG: control group; IG: intervention group; T: type; D: duration; F: frequency; I: intensity; PFM: pelvic-floor muscles; SAI: social activity index; IQR: interquartile range; SD: standard deviation; UI: urinary incontinence; #: number; DEC: duration of endurance contraction; MVC: maximum voluntary contraction; ICIQ-SF: International Consultation Incontinence Questionnaire—Short Form; UDI: Urogenital Distress Inventory; IIQ: Incontinence Impact Questionnaire; EMG: electromyographic; *: statistically significant.

## Data Availability

Not applicable.
